# Fetal cerebral ventriculomegaly: What do we tell the prospective parents?

**DOI:** 10.1002/pd.6266

**Published:** 2022-11-21

**Authors:** Veronica Giorgione, Karina Krajden Haratz, Shlomi Constantini, Roee Birnbaum, Gustavo Malinger

**Affiliations:** ^1^ Ob‐Gyn Ultrasound Unit Lis Maternity Hospital Tel Aviv Sourasky Medical Center Tel Aviv Israel; ^2^ Molecular and Clinical Sciences Research Institute St George's University of London London UK; ^3^ Sackler Faculty of Medicine Tel Aviv University Tel Aviv Israel; ^4^ Department of Pediatric Neurosurgery Tel Aviv Sourasky Medical Center Tel Aviv Israel

## Abstract

Fetal cerebral ventriculomegaly is a relatively common finding, observed during approximately 1% of obstetric ultrasounds. In the second and third trimester, mild (≥10 mm) and severe ventriculomegaly (≥15 mm) are defined according to the measurement of distal lateral ventricles that is included in the routine sonographic examination of central nervous system. A detailed neurosonography and anatomy ultrasound should be performed to detect other associated anomalies in the central nervous system and in other systems, respectively. Fetal MRI might be useful when neurosonography is unavailable or suboptimal. The risk of chromosomal and non‐chromosomal genetic disorders associated with ventriculomegaly is high, therefore invasive genetic testing, including microarray, is recommended. Screening for prenatal infections, in particular cytomegalovirus and toxoplasmosis, should also be carried out at diagnosis. The prognosis is determined by the severity of ventriculomegaly and/or by the presence of co‐existing abnormalities. Fetal ventriculoamniotic shunting in progressive isolated severe ventriculomegaly is an experimental procedure. After delivery, ventricular‐peritoneal shunting or ventriculostomy are the two available options to treat hydrocephalus in specific conditions with similar long‐term outcomes. A multidisciplinary fetal neurology team, including perinatologists, geneticists, pediatric neurologists, neuroradiologists and neurosurgeons, can provide parents with the most thorough prenatal counseling. This review outlines the latest evidence on diagnosis and management of pregnancies complicated by fetal cerebral ventriculomegaly.

## Key points

What's already known about this topic?

- Fetal ventriculomegaly is a relatively common finding on obstetric ultrasound examinations.
- The diagnostic workup includes genetic testing, infection screening, detailed anatomy ultrasound and neurosonography.
- The prognosis varies according to the severity of dilation and/or to the presence of other anomalies.

What does this review add?

- This review summarizes the most important aspects that an obstetrician should know in terms of diagnostic work‐up and prenatal counseling.
- The best prenatal counseling would be provided by a multidisciplinary fetal neurology team.

## 1 INTRODUCTION

**FIGURE 1:**
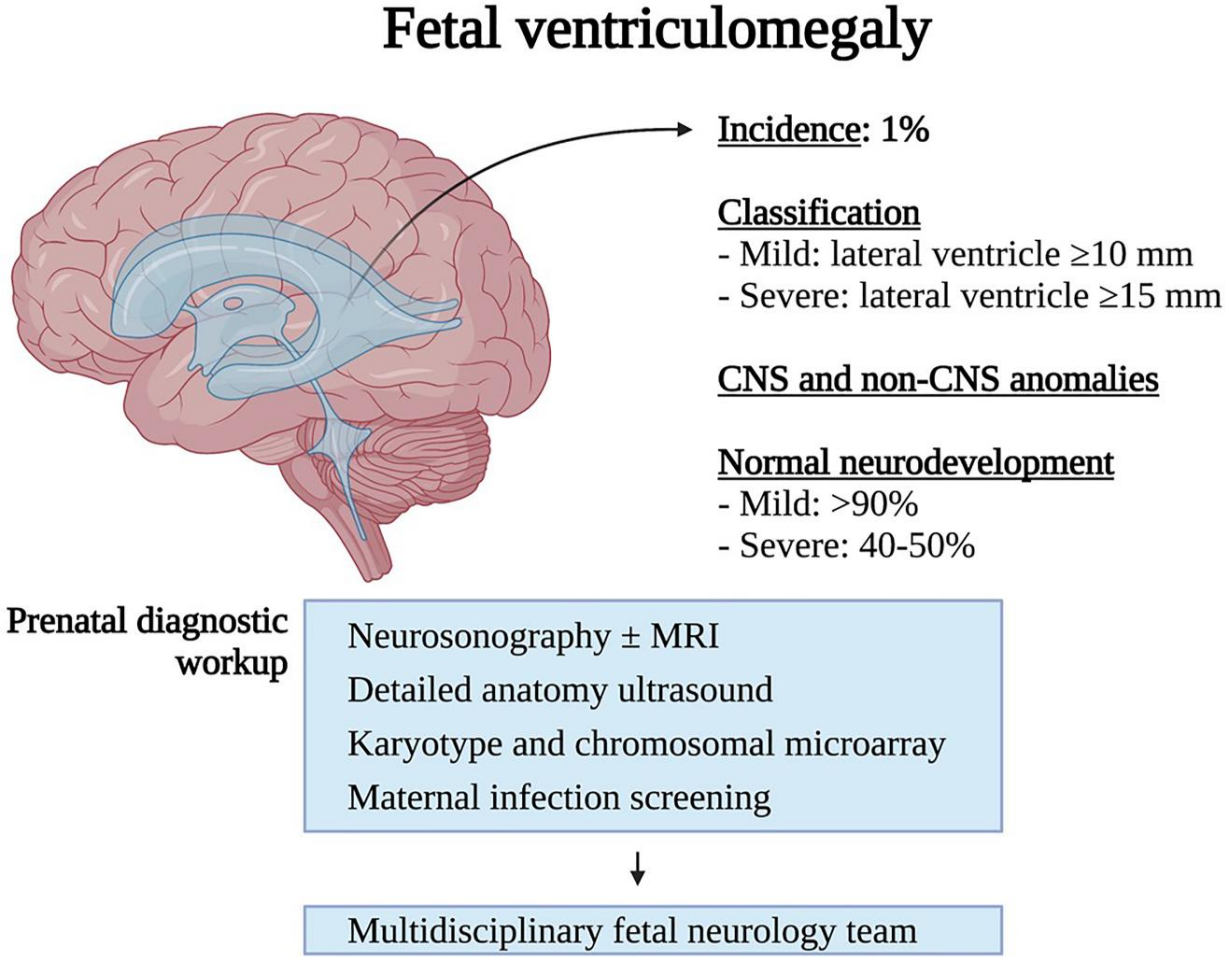
Fetal Ventriculomegaly. CNS, central nervous system

The study and measurement of the distal lateral ventricle is an integral part of the ultrasound (US) evaluation of the fetal anatomy that is routinely performed between 18 and 24 weeks gestation.^1^, ^2^ Ventriculomegaly is defined as a distal lateral ventricle measurement of 10 mm or more (Figure 1). Mild ventriculomegaly is diagnosed when the ventricle is less than 15 mm and severe when the ventricle is equal to or larger than 15 mm. During the third trimester and after a thorough neurosonographic evaluation, some authorities and our center consider that an isolated finding of ventriculomegaly with a measurement smaller than 12 mm and a head circumference normal or above the 50th centile is likely a normal variant and patients can be reassured.^3^, ^4^ Ventriculomegaly can be symmetric or asymmetric. Asymmetric ventriculomegaly is defined as a difference of more than 2 mm in the measurement with one or both ventricles larger than 10 mm. These definitions do not apply strictly to pregnancies during the first and early second trimester. Throughout pregnancy, ventriculomegaly might be stable, progressive, or regressive. In our experience, many cases showing a “decrease” in ventricular size are actually due to inconsistent adherence to proper technique or suboptimal imaging due to advanced gestational age.

Fetal ventriculomegaly can be an isolated finding or associated with other central nervous system (CNS) and non‐CNS anomalies, including genetic conditions that may not be evident at the initial evaluation or even after delivery.

## 2 EPIDEMIOLOGY AND RISK FACTORS

Fetal ventriculomegaly is quite common and present in around 1% of fetuses.^3^ Fetuses with extracranial anomalies, genetic conditions, or congenital infections are at increased risk of developing ventriculomegaly.

## 3 PRENATAL DIAGNOSTIC FEATURES

**FIGURE 2:**
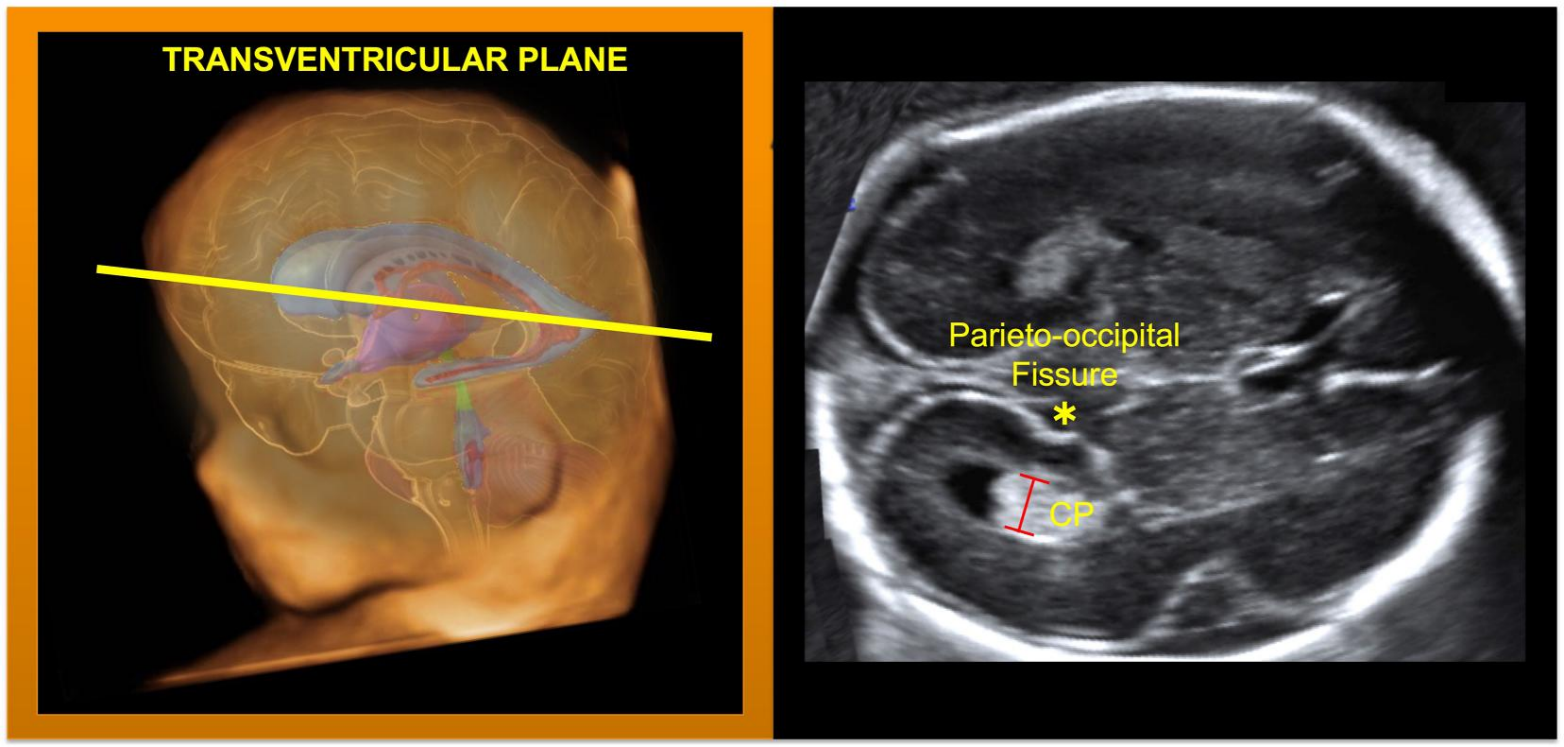
How to measure lateral ventricles from mid‐gestation

As previously mentioned, measurement of the atrium of the lateral ventricle is part of every fetal US anatomic screening and is performed in the axial transventricular plane at the level of the parieto‐occipital fissure (Figure 2). Usually, only the distal atrium is measured since optimal visualization of the proximal one can be technically challenging. In our experience, reassessment of the measurement before reaching a definitive diagnosis is a good practice since imprecision in measurement is common. Following confirmation of the diagnosis, a complete examination of the fetal anatomy, including a detailed neurosonographic assessment, preferably using the transvaginal approach if possible, is indicated to exclude as many as possible associated conditions (see Table 1). During the neurosonogram, the operator should assess the shape and the content of lateral ventricles (including the anterior horns), the morphology of their walls and the periventricular area to check for abnormal echogenicity, cyst or focal lesions. Moreover, the remaining parts of the ventricular system, midline structures, midbrain/hindbrain, brain parenchyma and sulcation, and neural tube should be assessed. When fetuses are in breech presentation, a gentle ultrasound‐guided external version may be considered if technically feasible.^5^ If the fetus remains in breech presentation, multiplanar US examination can be obtained by a fundal approach and by positioning the patient upright in the sitting position. Identifying associated abnormalities supports the fetal neurology team in determining the cause of ventriculomegaly and the prognosis.^6^, ^7^, ^8^

**TABLE 1:** Etiological classification of ventriculomegaly and associated CNS anomalies

Malformative
Holoprosencephaly
Agenesis of corpus callosum
Malformations of cortical development (lissencephaly, cobblestone malformations megalencephaly, schizencephaly, heterotopia/polymicrogyria)
Dandy‐Walker malformation
Obstructive
Intracranial tumor
Aqueductal stenosis
Midbrain‐Hindbrain malformation
Open neural tube defects‐Chiari Malformation Type 2
Clastic
Intrauterine infections
Ischemia/Stroke
Intraventricular hemorrhage
Atrophic
Metabolic disease
Neurodegenerative disorders
Blood‐stealing lesions following single fetal demise in monochorionic twin pregnancy

Abbreviation: CNS, Central nervous system.

## 4 DIFFERENTIAL DIAGNOSIS

It is essential to be aware that not only developmental brain anomalies can produce ventricular dilatation; acquired conditions such as infections, intraventricular hemorrhages and stroke need to be considered in the differential diagnosis, particularly when ventriculomegaly is detected late in pregnancy. The etiology of ventriculomegaly can be divided into malformative, obstructive, clastic, and atropic categories, as presented in Table 1.

## 5 RECOMMENDED PRENATAL INVESTIGATIONS AND WORKUP

### 5.1 Genetic

The most common chromosomal and non‐chromosomal genetic conditions associated with ventriculomegaly are displayed in Table 2.^9^

**TABLE 2:** Genetic conditions associated with ventriculomegaly

Genetic disorders	CNS findings
Chromosomal disorders	
Trisomy 21	Ventriculomegaly, holoprosencephaly
Trisomy 18	Ventriculomegaly, large choroid plexus cyst, cerebellar hypoplasia, mega cisterna magna, holoprosencephaly, dysgenesis of corpus callosum, spina bifida
Trisomy 13	Ventriculomegaly, cerebellar hypoplasia, mega cisterna magna, holoprosencephaly, agenesis of corpus callosum, microcephaly
Non‐chromosomal disorders	
X‐linked hydrocephalus	Ventriculomegaly, agenesis/dysgenesis of corpus callosum, adducted thumbs
Ciliopathies (e.g., Meckel‐Gruber syndrome*, Joubert Syndrome)	*Ventriculomegaly, occipital encephalocele, vermian and cerebellar hypoplasia, microcephaly, agenesis of corpus callosum, holoprosencephaly, anencephaly
Dystroglycanopathies (e.g., Walker‐Warburg syndrome*)	*Ventriculomegaly, malformation of cortical development, cerebellar anomalies, occipital cephalocele, agenesis/dysgenesis of corpus callosum, brainstem abnormalities (Z‐shape), eye anomalies
Tubulinopathies (e.g., TUBA1A)	Ventriculomegaly, malformation of cortical development, dysgenesis of basal ganglia, agenesis/dysgenesis of corpus callosum, cerebellar dysgenesis/hypoplasia, midbrain abnormalities
Others	‐

Abbreviation: CNS, Central nervous system.

Regardless of the severity of ventriculomegaly, the evaluation of apparently isolated cases should include genetic counseling regarding chorionic villus sampling or amniocentesis for chromosomal microarray.^4^, ^10^ In a cohort of 238 fetuses with CNS anomalies the prevalence of pathogenic DNA copy number variants (CNVs) detected with microarray was 6.7%, once common aneuploidies of chromosomes 13, 18, 21, X, and Y were excluded. Pathogenic CNVs were more frequent in cases complicated by posterior fossa anomalies and ventriculomegaly.^11^

Exome sequencing (ES), although currently not performed routinely, is likely to be used with increasing frequency in coming years.^12^, ^13^, ^14^ In multisystem malformations, the incremental yield of ES can reach 30%–33%.^15^, ^16^ In major CNS anomalies the diagnostic yield of ES has been reported around 19%–44%, whereas in isolated, single, CNS anomalies, 7.2% of fetuses had a likely pathogenic/pathogenic variant at ES including 3 out of 23 (13%) fetuses with isolated mild ventriculomegaly.^17^, ^18^ However, the application of ES in prenatal diagnosis has significant challenges, including the requirement for highly specialized expertise, limited data on fetal phenotype‐genotype correlations, and ethical issues including incidental and uncertain findings in fetuses and/or parents. Therefore, such advanced genomic testing should only be offered by centers with the appropriate resources and expertise.^14^ Moreover, a pre‐test genetic consultation is warranted if such testing is considered.

### 5.2 Infection

Evaluation for possible intrauterine infection or exposure to teratogenic substances is also recommended in the workup of ventriculomegaly.^19^, ^20^ The prevalence of infections in fetuses with ventriculomegaly is about 1.4%, with the most common infective agents being cytomegalovirus and toxoplasmosis.^20^ Other rarer viral infections, such as rubella, Parvovirus B19, Zika and Herpes simplex virus could result in ventriculomegaly and should be investigated in women with identified risk factors.^19^, ^21^, ^22^, ^23^ Ventriculomegaly due to an infectious etiology is usually accompanied by other CNS and extra‐CNS findings. Although performing polymerase chain reaction on amniotic fluid is the most reliable test, maternal serology can be carried out as a first‐level infection screening. A serology result that is negative for both IgG and IgM antibodies effectively excludes an infection as a differential diagnosis. Avidity testing and retrospective testing of stored serum samples from early pregnancy (if available) may be required for interpretation of serology results and estimating the timing of possible maternal infection.

### 5.3 Fetal MRI

It is controversial if a fetal MRI should be performed in all cases complicated by ventriculomegaly.^24^, ^25^ Following a good quality neurosonographic study, fetal MRI will add clinically relevant information in only a small percentage of cases.^26^, ^27^ Fetal MRI detected additional associated anomalies in 5% (95% CI 3.0–7.0) of fetuses with mild or moderate ventriculomegaly assessed by a dedicated neurosonography, but in 16.8% (95% CI 8.3–27.6) of fetuses assessed by a standard ultrasonography.^27^ When dedicated neurosonography is unavailable or technically difficult to perform, MRI performed during the third trimester by qualified operators has proved to be helpful.^28^, ^29^, ^30^ It is important to know that a slight increase (around 1 mm) in the width of the ventricle can occur when measured by fetal MRI as compared with ultrasound.^31^

After 3–4 weeks, a follow‐up neuronosonographic examination or fetal MRI (in the above‐mentioned cases) are essential to complete the work‐up because of possible progression of ventriculomegaly and/or development of other CNS malformations.^32^, ^33^

## 6 PROGNOSTIC FACTORS

The main factors affecting prognosis are the severity of the ventricular dilatation, progression during pregnancy or following delivery, a head circumference smaller than expected for the gestational age (not necessarily microcephaly), growth retardation, abnormal genetic or infectious tests, and the presence of associated anomalies.^33^ Moreover, a worse prognosis is expected when there is an early onset (first or early second trimester) of severe ventriculomegaly during pregnancy.^34^ Further details on postnatal outcomes are discussed below.

## 7 OPTIONS FOR THERAPY

### 7.1 Fetal surgery

Intrauterine ventriculoamniotic shunting is an experimental procedure for severe ventriculomegaly and is sometimes considered to minimize maternal or fetal morbidity in ongoing pregnancies. The procedure needs to be carried out in tertiary centers with expertise in the field.

When the first procedures were attempted in the 1980s, they were associated with an intra‐procedure fetal death rate of 10% and moderate‐to‐severe disability in 66% of survivors.^35^ Therefore, these results did not show an improvement in outcome over expectant management and the procedure was abandoned. In a contemporary case series from 2019 of 44 fetuses with bilateral severe ventriculomegaly (>20 mm), the intraprocedural death rate was 7%. The moderate‐to‐severe neurodevelopmental delay was reported in 30% of those with isolated ventriculomegaly and 82% of those with non‐isolated ventriculomegaly.^36^ In view of the lack of substantial improvement in infant outcomes reported in this more recent experience, the role of fetal surgery remains controversial. Some investigators continue to support its consideration for selected cases, such as progressive, isolated severe ventriculomegaly of non‐infectious obstructive origin, primarily aqueductal stenosis.^37^, ^38^ Randomized studies are necessary to assess the potential long‐term benefits on the neurodevelopmental outcome.

### 7.2 Postnatal surgery

The indication for, and timing of, postnatal surgical interventions in infants with ventriculomegaly depend on several factors, including the presence of symptoms (e.g., headaches, vomiting, irritability, developmental delays, focal neurologic deficits, papilledema), progression of the ventriculomegaly, or evidence of obstruction of the cerebrospinal (CSF) pathway on postnatal imaging. Two different therapeutic options are possible, ventricular‐peritoneal shunting or ventriculostomy. The former is performed by inserting a tube that connects the ventricular system to the peritoneum where CSF is absorbed, bypassing the site obstruction. Ventriculostomy consists of creating an opening at the base of the third ventricle, using a minimally invasive approach. The overall health and quality of life at five‐year follow‐up in children treated for hydrocephalus caused by aqueductal stenosis were not different between the two methods.^39^

## 8 IMPACT ON TIMING AND MODE OF BIRTH

### 8.1 Mild ventriculomegaly

The timing and route of delivery should be based on standard obstetric indications, in particular when the head circumference is normal.^4^

### 8.2 Severe ventriculomegaly

In severe ventriculomegaly, there is no evidence that an early postnatal shunting can improve the prognosis and the well‐known risk related to prematurity should be avoided. Moreover, delivery less than 40 weeks has been associated with an increased risk of postnatal shunt failure.^40^ In severe ventriculomegaly, the cesarean section rate has been reported to be high (>50%). For instance, in a cohort of 72 pregnancies complicated by severe ventriculomegaly (15–20 mm or >20 mm) and macrocrania (head circumference >95th percentile for gestational age), the cesarean section rate was 65.3%.^41^ For fetuses with macrocephaly, planned cesarean delivery might be the best option when pelvic‐cephalic disproportion is likely during labor. The use of cephalocentesis is extremely rare and could be reserved for cases of guarded fetal prognosis, to reduce maternal morbidity.^42^

In conclusion, delivery plans for fetuses with macrocephaly and severe ventriculomegaly should be customized to improve neonatal outcomes when possible and reduce risks to the mother.

## 9 EXPECTED RANGE OF POSTNATAL OUTCOMES

Parents should understand that fetuses with apparently isolated ventriculomegaly in utero might have other structural abnormalities on examination after birth. The false‐negative rate of prenatal imaging is 7.4% and 9.5% in apparently isolated fetal mild and severe ventriculomegaly, respectively. The most common anomalies found on postnatal imaging were malformations of cortical development.^43^, ^44^ Moreover, they should consider that reported data on developmental delay might vary among studies because of different neurodevelopmental evaluations, length of follow‐ups, and inclusion or exclusion of fetuses with additional anomalies. When the cause of ventriculomegaly has been identified, the parental counseling can be more precise than ventriculomegaly with unknown etiology.

### 9.1 Mild ventriculomegaly

Survival rate in mild to moderate apparently isolated ventriculomegaly is 97%–98%, and normal neurodevelopment is expected in more than 90% of these children.^6^, ^44^ Therefore, mild and even moderate isolated ventriculomegaly is usually associated with a normal outcome, in particular, if unilateral.^45^, ^46^, ^47^ In a 2014 meta‐analysis including 652 cases of isolated mild ventriculomegaly with postnatal follow‐up, the overall prevalence of developmental delay was 7.9% (95% CI 4.7%–11.1%) at a median age of 30 months.^43^

### 9.2 Severe ventriculomegaly

Severe ventriculomegaly is associated with a survival rate of 88% and with neurologic, motor, and cognitive impairment in around 60% of these children.^48^ The neurodevelopmental outcome is worse when other CNS or non‐CNS anomalies are present and the prognosis depends on the type of anomalies. Moreover, another poor prognostic finding is the persistence or progression of ventriculomegaly.^44^, ^49^, ^50^

## 10 MULTIDISCIPLINARY TEAM INVOLVEMENT

The diagnosis of a suspected pathology involving the fetal brain, particularly when the expected prognosis is uncertain, requires a great deal of knowledge and tactful counseling of parents. Moreover, prenatal counseling might be challenged by the possibility of a diagnosis of CNS abnormality only in the third trimester due to the continuous development of fetal brain throughout gestation.

**FIGURE 3:**
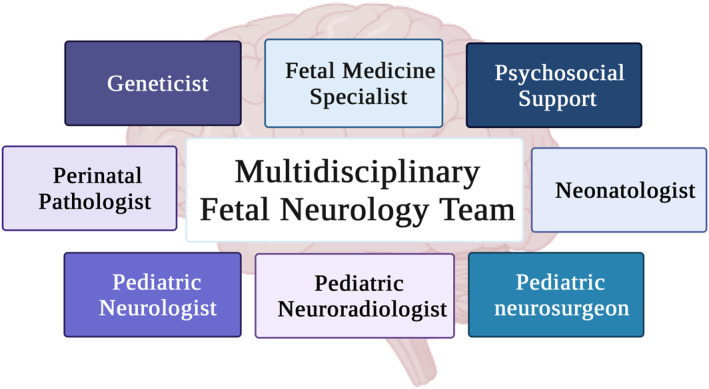
Multidisciplinary fetal neurology team

In our experience, parents are often confused and anxious when a suspicion of a fetal brain anomaly is raised. Therefore, communicating a wealth of information at the first consultation can be challenging. Due to this fact, a second planned visit, this time with the participation of relevant members of the multidisciplinary team, is indicated (see Figure 3).^51^ In cases of severe isolated ventriculomegaly, consultation with a pediatric neurosurgeon is advisable. In patients with mild ventriculomegaly, we convene the pediatric neurologist, the genetics expert and the psychosocial team; when an MRI is performed, the pediatric radiologist is also invited.

In ideal conditions, the whole team participates in the meeting with the family; when this is not possible, the meeting takes place as part of the weekly discussion of cases with suspected anomalies and the parents are informed of the recommendations later on.

## 11 FUTURE RECURRENCE RISK

The risk of recurrence depends on the etiology; whenever the etiology is not revealed following a complete investigation, the risk of recurrence is low.^52^

## 12 CONCLUSIONS

Fetal cerebral ventriculomegaly is a common prenatal finding during fetal US evaluation. The prognosis depends on the etiology and on the presence of other CNS and extra‐CNS anomalies. Parents should be offered further investigations throughout pregnancy, considering the dynamic development of the brain during fetal life. A multidisciplinary fetal neurology team can provide comprehensive information and support parents in making informed decisions.

## CONFLICT OF INTEREST

Gustavo Malinger is a member of the Editorial Board of Prenatal Diagnosis. The other authors have no conflict of interest to declare.

## Data Availability

Data sharing not applicable to this article as no datasets were generated or analyzed during the current study.
